# Recommendations for marathon runners: on the application of recommender systems and machine learning to support recreational marathon runners

**DOI:** 10.1007/s11257-021-09299-3

**Published:** 2021-08-18

**Authors:** Barry Smyth, Aonghus Lawlor, Jakim Berndsen, Ciara Feely

**Affiliations:** Insight SFI Centre for Data Analytics, University College Dublin, Dublin, Ireland

**Keywords:** Recommender systems, Marathon running, Personalised fitness

## Abstract

Every year millions of people, from all walks of life, spend months training to run a traditional marathon. For some it is about becoming fit enough to complete the gruelling 26.2 mile (42.2 km) distance. For others, it is about improving their fitness, to achieve a new personal-best finish-time. In this paper, we argue that the complexities of training for a marathon, combined with the availability of real-time activity data, provide a unique and worthwhile opportunity for machine learning and for recommender systems techniques to support runners as they train, race, and recover. We present a number of case studies—a mix of original research plus some recent results—to highlight what can be achieved using the type of activity data that is routinely collected by the current generation of mobile fitness apps, smart watches, and wearable sensors.

## Introduction

Recommender systems influence our media consumption (books, movies, music, news), shopping habits (online and real-world), and even the people we interact with every day (Ricci et al. 2015; Bridge et al. 2005; Smyth 2007; Burke 2002). Their success has not been without its challenges, especially as we have come to understand the associated privacy and ethical issues that arise as a result of their widespread application and adoption (Lam et al. 2006; Rooksby et al. 2014; Knijnenburg and Kobsa 2013). If we can address these challenges, then there is the potential for recommendation technologies to bring valuable insights to bear on many aspects of our lives (Kelly et al. 2013) and to the societies we live in, by helping to nudge us in the direction of a healthier, happier, and more sustainable way of living.

This is especially true in the area of personal health (Mayer-Schönberger and Cukier 2013), and over the past decade, mobile devices and wearable sensors have proven to be important enablers when it comes to supporting people in their efforts to adopt healthier, and more active lifestyles (Mulas et al. 2011; Pilloni et al. 2013; Mulas et al. 2013; Dunne et al. 2008, 2007; Dunne and Smyth 2007). For example, mobile apps like *couch-to-5k*^1^ have helped to encourage millions of people to begin their fitness journey, while the likes of TrainingPeaks^2^, Strava^3^ and RunKeeper^4^ have helped millions more to stay motivated, remain active, and become even fitter (Schoeppe et al. 2016; Lister et al. 2014; King et al. 2013; Sundar et al. 2012; Vickey et al. 2012; Sullivan and Lachman 2017; Hosseinpour and Terlutter 2019; Pilloni et al. 2018; Boratto et al. 2017; Direito et al. 2017; Zhao et al. 2016; Vandelanotte et al. 2016). In the main, these apps are focused on helping users to record and review their exercise habits. They track steps, distance, and speed, and provide insights into progress and goals. They even help to connect friends and like-minded individuals into supportive social networks that can help people to stay motivated and engaged.

**Fig. 1:**
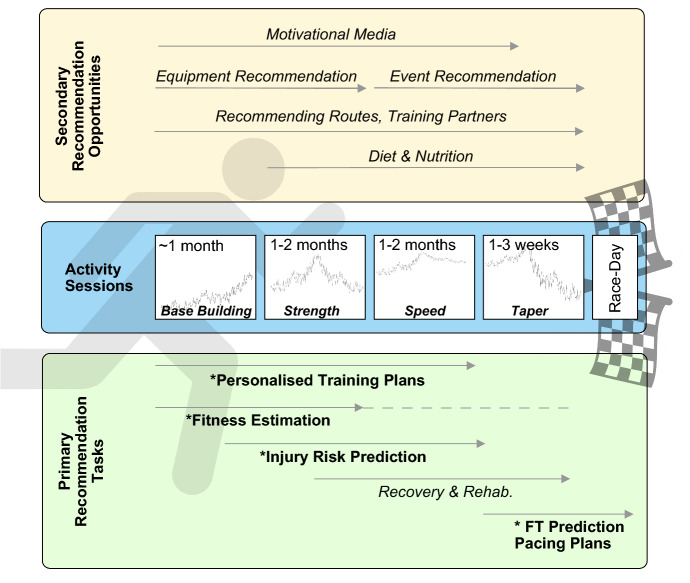
A vision for supporting runners using recommendation techniques as they train for endurance events such as the marathon. A typical runner will engage in at least 4 months of training activity as they move through specific training periods such as building a suitable fitness base (approximately 4–6 weeks), strength/endurance training (1–2 months), speed training (1–2 months), and finally a period of more gentle training (the so-called *taper*) in the final 1–3 weeks before race-day; generally speaking, the periods associated with developing strength and speed may overlap, at least in part. This generates dozens of individual training sessions with different physiological objectives depending on the training period. Alongside this training, there are many training-specific (primary) and secondary recommendation opportunities as shown; those marked with an ‘*’ are the subject of case studies in Sects. 4–7

We believe that activity data have the potential to tell us not just about how we *have* been exercising, but also how we *should* be exercising, to get the most from the activities we engage in. With this in mind, in this work we focus on bringing recommendation techniques to recreational endurance athletes—marathon runners in particular—to help them to train, compete, and recover more effectively and more safely; see also (Cheung et al. 2019). To achieve this, in Fig. 1 we present a particular vision for the role of recommender systems in helping people as they prepare for, participate in, and recover from, marathon races; although this provides a marathon specific perspective, it should be easily adapted for a wide range of structured, endurance activities. At the heart of this vision are the *activity sessions* that runners engage in as they train, typically for at least 3–4 months before race-day. Different training periods focus on different types of physiological adaptations, such as building an initial base of fitness, improving strength, increasing speed, and finally, *tapering* to recover before race-day. All of this training must be carefully coordinated so that a runner achieves peak fitness just before race-day while minimising their risk of injury.

This vision helps to clarify two ways in which recommender systems can play an important role when it comes to supporting the many and varied needs of marathon runners as they train. First and foremost, and the main focus of the technical work presented in this paper, are the *primary* recommendation tasks that are designed to help runners with their training, from recommending personalised training programmes all the way to helping runners to plan their race by recommending suitable pacing strategies and achievable finish-times. In Fig. 1 a number of these tasks are highlighted because they will form the basis of a series of four case studies in Sects. 4–7 of this paper. Two of these case studies—fitness estimation (Sect. 4) and predicting injury risk (Sect. 6), cover original research that has not been previously published. The other two case studies, training plan recommendation (Sect. 5 and Feely et al. 2020b, a) and finish-time prediction (Sect. 7 and Smyth and Cunningham 2017b, 2018b, 2017a, 2018a), have been published previously, but are reproduced here in summary form in order to provide end-to-end examples of some of the ways that machine learning and recommender systems can support runners, at different stages during their physical training, from the moment they begin their marathon journey through to race-day itself. The hope is that this broader set of case studies will serve as a useful catalyst for similar lines of work in the future.

In addition to these primary recommendation opportunities, Fig. 1 highlights a number of secondary recommendation opportunities, many of which share a close affinity with more conventional e-commerce tasks (e.g. equipment/gear or event recommendations) or media-related recommendation tasks (e.g. suggesting relevant articles, videos about training objectives or recommending motivational podcasts to take on a run), or even social recommendation tasks (such as, recommending like-minded, training partners with similar ability levels). These secondary tasks serve to further highlight the scope of recommendation opportunities that exists in this domain.

The remainder of this paper is organised as follows. In the next section, we present a comprehensive account of the marathon as a novel recommendation domain, explaining why we believe this to be the case and summarising the many and varied recommendation and machine learning opportunities that are highlighted in Fig. 1, along with relevant existing research. Following this we present an overview of the case studies that are included in this work, focusing on the main research questions they are designed to answer, and summarising the datasets used. After this, we present the four individual case studies, presenting their main objectives, the approach taken, and their key results, as well as a discussion of their main limitations. Finally, we conclude by summarising the main results and offering opportunities for future research.

## The marathon as a novel recommender systems domain

Marathons make for an interesting domain for recommender systems and machine learning research for several different reasons (Smyth 2019):

1. There exists a large community of highly motivated, yet often inexperienced users, who are actively seeking out advice and guidance on a variety of topics, from training and injury prevention, to equipment recommendations and race planning. For example, a significant proportion of marathon runners (perhaps 30–50% depending on race) are first-timers and as such are among the most needy when it comes to training advice, and the most at risk when it comes to training-related injuries. They are an ideal target audience for recommender systems, but so too are experienced runners, whether they want to improve their finish-times, extend their running-life by training more carefully as they age, or simply want to find new challenges and friends to share them with.
2. Runners generate a plentiful supply of detailed activity data—from fine-grained training activities (distance, pacing, heart-rate, etc.), to rest/recovery data, race results and time-trials, even nutrition—which can be harnessed to better understand their abilities, preferences, and goals. These days most people training for a marathon will use an app like Strava or Runkeeper perhaps in conjunction with a fitness-related smartwatch or other sensors to measure heartrate, power, running economy, etc. That being said, the sheer quantity of data, and the level of detail and precision available, makes it challenging to deal with. These data are far from perfect, because of issues such as GPS errors, the varying accuracy of off-the-shelf heartrate sensors, etc., which introduces some challenging feature extraction issues.
3. As mentioned in the previous section, training for, competing in, and recovering from, the marathon, encompasses a wide variety of recommendation tasks and opportunities, some familiar, some less so. All of these opportunities can benefit from a novel approach to user profiling, which relies on a mixture of activity and physiological data, as well as more conventional ratings and preferences. Moreover, the ubiquity of mobile devices means that there are new opportunities for delivering real-time, multi-modal (visual, audio, haptic) interventions to help runners cope with the challenges they face.

In what follows we will summarise these opportunities in more detail by reviewing related research according to the framing of our vision in Fig. 1. This is not intended to provide a complete *systematic review* of the literature, which is beyond the scope of this paper, but rather to bring together key ideas and results from relevant research as it relates to the recommendation opportunities highlighted in Fig. 1. We will do this by first discussing the primary recommendation opportunities that are related to the physical aspects of marathon training, followed by a review of the secondary recommendation opportunities, which may be more familiar to many in the recommender systems community.

### Primary recommendation opportunities

In this section, we will review several open challenges in the exercise physiology and sports science communities (Fawcett 2015; Millington 2014; Panjan et al. 2010; Cornforth et al. 2015; Maier et al. 2018; Akay et al. 2017; Zhang 2019; Taha et al. 2018; Yingying et al. 2010; Whiteside et al. 2017; Abt and Lovell 2009; Jelinek et al. 2014). While previous efforts have largely focused on physiological modelling, laboratory protocols, and *elite* athletes, our main focus will be to highlight how to help *recreational* runners by applying machine learning and recommender systems techniques to the type of activity data that is routinely generated when we train.

#### Fitness estimation and training effects

Sports scientists use a variety of important laboratory metrics to estimate the fitness levels of individuals and how they change under different training conditions. The well-known $VO_{2}max$ score (Noakes 2003; Daniels 2013; Billat et al. 1994) measures the maximum rate of oxygen consumption during exercise. It reflects the cardiorespiratory fitness of an individual and is an important determinant of their endurance capacity during prolonged exercise. It is usually measured in a laboratory setting and, as such, is not routinely accessible by recreational athletes.

With the advent of smartwatches and wearable sensors, however, it is now possible to estimate $VO_{2}max$ based on training effort under specific conditions. For instance, there are a number of examples of recent research on the use of machine learning for $VO_{2}max$ prediction (Akay et al. 2011; Abut et al. 2016) but many challenges remain to improve prediction accuracy under real-world, recreational training conditions. These have the potential to provide accurate $VO_{2}max$ estimates without the need for expensive laboratory support; see also (Webb et al. 2014; De Brabandere et al. 2018; Akay et al. 2013, 2009). Similar approaches can to be applied to predict other key performance metrics, such as a runner’s *lactate threshold*^5^, or the *training effect* of a specific session, which can be used as a measure of fitness improvement.

All of these estimation problems can be readily framed as classical supervised learning tasks. The resulting models have the potential to transform the effectiveness of training programmes by accommodating the provision of core, targeted, personalised advice and tailored recommendations to an athlete on any given day. In particular, the availability of such fitness-related features will help recommender systems to deliver more relevant recommendations and more accurate suggestions when it comes to recommending appropriate training sessions or challenging but achievable target race-times, and in Sect. 4 we discuss our own attempts to generate accurate fitness estimates using the type of activity data routinely collected by runners as they train.

#### Training session classification

When it comes to training, runners and cyclists talk in terms of *intervals*, *hill-repeats*, *tempo sessions*, *threshold training*, *fartleks*, *easy-days*, *progressions*, *ladders*, *speed-work*, *yasso-800s*, etc. These are all different types of training sessions, designed to promote specific training effects. For example, running interval sessions, where the runner alternates between periods of fast running (for 400 m, 800 m or 1500 m distances) and recovery, can improve aerobic and anaerobic endurance, increase $VO_{2}max$, and improve overall performance, while the increased *‘afterburn’*—referring to post-exercise calorie consumption—can aid in weight-loss.

While training programmes prescribe a variety of different session-types, current apps do little when it comes to monitoring or assessing an athlete’s adherence to specific training sessions, and they are far from being able to recommend specific sessions in all but the most limited of contexts. Most apps simply do not have any understanding of the nature or purpose of such sessions. They record GPS, pacing, and heartrate traces without encoding any of the key features that distinguish different sessions. This makes for a significant opportunity to draw on recent work about detecting structures and motifs in time-series (Senin et al. 2018; Berlin and Laerhoven 2012; Cheng 2013) in order to: (a) automatically classify training sessions to better assess a runner’s performance or fitness level and, in due course, to adapt their training programme appropriately; and (b) to dynamically assess how well an individual is adhering to a particular training session, to provide in-session feedback (increase/decrease interval pace, reduce/extend interval duration, adjust recovery period, etc.) and guide the individual to a better session outcome.

The ability to classify a training session, combined with accurate models of fitness and training effort, will make it possible to provide an individual with more targeted advice about the effectiveness of their training as well as pinpointing areas for improvement. This will help a runner understand whether they have pushed themselves too hard, or not hard enough, for instance, and can provide the basis for adaptations to a training programme to better balance activity and recovery.

#### Injury prediction and training load estimation

Indeed, recovery is a critical, but all too often overlooked part of any training programme. Recovery days allow the body to adapt to training and to replenish vital resources (Noakes 2000). Insufficient recovery can lead to missing out on fitness gains, and keeping track of recovery levels can reveal when a hard training period is likely to be beneficial or injurious to an athlete. An important opportunity exists to estimate recovery needs, based on an athlete’s current fitness levels, recent training effort, and key physiological indicators such as resting heartrate. While some fitness devices do include some recovery estimation features, they tend to be simplistic and offer considerable room for improvement (Pulkkinen and Saarikoski 2010). In the future, athletes will benefit from more insightful and actionable recovery recommendations, not only about how long they should recover for, but also about *how* they should recover and the type of activities they should and should not engage in Glaros et al. (2003).

A related matter is *training load*, which provides a big-picture estimate of an athlete’s current training effort, and can be an important indicator of common problems such as over-training (Thornton et al. 2017; Malisoux et al. 2015; Lazarus et al. 2017; Barros et al. 2017; Bowen et al. 2019); although it is not without a level of controversy, see, for example, (Bornn et al. 2019). Activity data provide a rich source of training data for machine learning, by integrating fitness and physiology data with training volumes, and user-provided training assessments, e.g. by logging effort perceptions, documenting injury and illness. In due course, it may be possible to identify novel patterns linking fitness, training, recovery and injury and so develop effective early warning systems for athletes, to alert them to changes in their performance, which may be a precursor to the onset of illness or injury (Gabbett 2016; López-Valenciano et al. 2018; Claudino et al. 2019). Accurately predicting whether a runner will become injured, or is at greater risk of injury, is an extremely challenging task (Carey et al. 2017; Kampakis 2016; Rossi et al. 2018), and we describe some early efforts in this regard in Sect. 6.

#### Personalised training programmes

Perhaps the holy grail for recreational endurance athletes is the ability to benefit from *personalised training programmes* tailored to the precise needs and preferences of an individual; their preferred training load, types of sessions, duration, etc. Most recreational athletes train using some form of training programme, usually one that they have found online, or one that they have adapted to their own needs over the years. These programmes will typically break a 12–16 week training period into a number of 3–4 week blocks, with each block made up of a number of specific training sessions in order to produce a given training effect (e.g. strength, endurance, etc.); see Fig. 1 and (Fry et al. 1992a, b). Programmes may also include specific rest, recovery and dietary components.

In the first instance, it can be challenging for an individual to find a training programme that suits their particular personal circumstances and goals, and many are left struggling to follow a mismatched, *one-size-fits-all* programme. Recently the concept of a *virtual coach*, capable of offering more personalised training advice, has been proposed in the literature (Fister et al. 2015; Rauter 2018) for resistance training and mountain biking; see also (Loepp and Ziegler 2018; Ni et al. 2019). Similar ideas may be suitable to develop personalised programmes for other endurance athletes, by harnessing accurate, real-time, personal measures of an individual’s fitness, physiological well-being, training load, etc.

The challenge in creating appropriate training programmes, which are fine-tuned to an individual’s needs, is that it requires a deep understanding of human physiology and the specific demands of the marathon as it relates to training. Thus, producing a multi-session, multi-week plan that suits a given runner is a significant planning and recommendation challenge, compared to the more typical item-based recommendations. In principle, personalised training plans can be generated by matching particular training needs with specific training sessions to provide the individual with specific guidance about how to conduct these sessions in terms of pace and intensity. Of course how an individual responds to a given session, or training block, can be used to fine-tune future sessions or re-plan as needed. Later in Sect. 5 we discuss an example of this in the form of a case-based reasoning (CBR) approach to personalised training, based on the work of Feely et al. (2020b, 2020a), but other approaches may also be relevant including ideas from more traditional planning and plan adaptation research (Munoz-Avila and Cox 2008; Hanks and Weld 1994).

Personalised training recommendations can, and should, also be augmented with supporting explanations so that the athlete can better understand the reason why a specific session is being recommended, how they should approach it, and how they should recover afterwards to gain maximum benefit. There is a growing body of research on the topic of *explainable AI* which has the potential to play an important role in this regard; see, for example, (Shin 2021).

#### Goal-time prediction and pacing

So far we have discussed supporting individuals during training, but of course all of this training will be in the service of a specific event, or set of events, such as an upcoming marathon or triathlon. As an event approaches, an individual will start to plan their goal-time and race strategy. In endurance events, such as the marathon or triathlon, it is not enough to aim for a specific goal-time, it is just as important for participants to plan how to pace their race on the day, including their in-race nutrition to fuel their efforts (Jeukendrup 2011), strategies to avoid hitting the wall, etc. (Buman et al. 2008; Smyth 2018; Ely et al. 2008; Doherty et al. 2020).

When it comes to goal-time prediction, there is a body of work that uses linear models to predict future race-times based on previous race-times, e.g. (Bartolucci and Murphy 2015; Schmid et al. 2012). What is less well developed, however, is the translation of a goal-time into a specific race strategy, and a concrete set of pacing recommendations. We have recently addressed this dual problem of goal-time prediction and pacing recommendation for marathons (Smyth and Cunningham 2017b, 2018b, a); we summarise these efforts in Sect. 7. Briefly, the goal-time and pacing plan for a target runner is adapted from the race-times and pacing profiles of runners with similar race histories. Recent research (Smyth and Cunningham 2018a) has considered different representations to encode the marathon history of runners and their impact on goal-time prediction accuracy and pacing-plan quality, demonstrating that accurate predictions, and high-quality pacing-plans, can be generated for all levels of runner ability; we will return to this topic in more detail in our final case-study later in this paper. Similar approaches can also readily adapted for other forms of endurance sport (McConnell and Smyth 2019; Smyth and Willemsen 2020), and these methods have the potential to offer valuable pre-race advice and even in-race guidance (Berndsen et al. 2019) to individuals.

### Secondary recommendation opportunities

As mentioned already, there are many other interesting recommendation and machine learning opportunities aside from those directly connected to the physical aspects of training and competing. In particular, helping to maintain a runner’s interest during the long weeks of training is a major motivational challenge that existing recommendation techniques may be well suited to support, for example, by helping to motivate a runner by suggesting relevant media (online articles, training videos, etc.), or by making training a more social activity by suggesting suitable training partners, or by proposing new routes to explore, all of which are familiar recommendation tasks. But there are more unusual and more speculative open recommendation opportunities too, such as how recommender systems might be able to help runners to rest and recover more effectively by suggesting suitable recovery activities or by suggesting healthier sleep habits.

#### Event recommendation

Recommending events (marathon-distance or other distance running events) to a runner during their training, by considering the target event they are training for, where they are in their training programme (fitness and training load), the location of the event, course terrain and other conditions, etc., can be an important way to supplement their training. There are many examples of event recommendation techniques in the literature for various event types and they could be readily adapted for marathon runners (Macedo et al. 2015; Minkov et al. 2010; Qiao et al. 2014). By selecting the right event at the right time a recommender system can add significant value to a runner’s training—pushing the runner to achieve new performance and fitness goals—as well as populating long training programmes with interesting and enjoyable intermediate events. Indeed, recommending a race event may also encourage a runner to test their current fitness level, by running at close to their maximum ability, which in turn may help produce the type of data that is needed for more accurate fitness assessments.

#### Recommending routes and training partners

In the past recommender systems have been used in route planning (McGinty and Smyth 2001; Chakraborty 2012), particularly in tourism applications (Gavalas et al. 2014; Borràs et al. 2014; Werthner and Ricci 2004; Ricci 2002) to recommend interesting routes for users to follow as they explore a new location. Similar ideas may be useful when it comes suggesting training routes to runners, especially if they have to travel to a new location during training. And by extracting useful information about a runner’s preferred home routes (e.g. terrain, elevation, distance, difficulty, distance from home, etc.), it may be possible to use content-based recommendation techniques to identify routes with similar characteristics to a travelling runner. Indeed, such a task may also be amenable to more conventional recommendation techniques in the form of *“runners like you have also run the following routes ...”*, based on features that describe types of runners (age, ability, training frequency, training distances, etc.).

Moreover, since running can be a social activity it can be useful to recommend running partners, perhaps based on availability, ability, or the target training session, but perhaps also based on broader interests so that the conversation flows during longer, easy runs; see, for example, related ideas in Goyal et al. (2018), Kurade (2014), O’Donovan et al. (2008), O’Donovan et al. (2009), Tang et al. (2013). Indeed, this speaks to an obvious connection with so-called *reciprocal recommender systems* (*RRS*). A reciprocal recommender is a type of recommender system that can be distinguished for tasks in which people are both the subjects and objects of recommendation (Li and Li 2012); for example, in a job recommendation setting, jobs may be recommended to people (people are the recommendation *subjects*) or people may be recommended for jobs (people are the *objects* of recommendation) (Hong et al. 2013; Ding et al. 2016; Li and Li 2012). Other examples of RRS include matching students with shared interests (Prabhakar et al. 2017) and online dating (Pizzato et al. 2010b, a; Akehurstet al. 2011). And ideas from the RRS literature provide a useful perspective when it comes to suggesting well-matched training partners, In particular, reciprocal recommender systems explicitly consider the importance of matching *reciprocity* (a successful match depends on a mutual, two-sided preference) as well as the *availability* of users, and the *passiveness* of users, all of which play an important role in the overall quality and health of a reciprocal network; see (Li and Li 2012) for further discussion.

#### Gear and equipment recommendation

Although running places a relatively low equipment burden on a runner—most require little more than shoes, shorts, and a top—pairing the right equipment with the right runner is extremely important, especially when it comes to footwear. Quite simply, the wrong running shoes can sabotage a runner’s training by increasing the likelihood of injury (Ryan et al. 2011). Of course recommender systems have a long history when it comes to suggesting clothing to users and by incorporating information about a runner’s physical characteristics (sex, weight, age, gait) and their training (weekly volume, pace, terrain, etc.), it should be possible to make highly targeted and appropriate footwear recommendations to optimise training and racing, and reduce the risk of injury or discomfort; see, for example, (Marks 2017; Zrenner et al. 2018; Frejlichowski et al. 2016; Wakita et al. 2015; Hwangbo et al. 2018). Beyond footwear recommendation, similar opportunities exist when it comes to suggesting other forms of equipment, such as clothing that is well suited to climate and distance, for example.

#### Nutritional support

Training for a marathon is not just about mastering the miles, it is also about fuelling the miles, and all too many runners make their training harder than it should be by ignoring the nutritional aspects of training. The same is even more true on race-day: getting your in-race nutrition wrong can be the difference between the elation of a new personal-best and the agony of hitting the wall. Thus, there is a need to support runners with targeted advice about their nutritional needs, preferably by paying attention to their food preferences and current diet. Recommender systems have form when it comes to providing this type of advice (Mika 2011). For example, the works of Ge et al. (2015), Ribeiro et al. (2017), Khan et al. (2021) have used ideas from recommender systems research to guide people towards more healthy eating habits by recommending more balanced and healthy meal-plans. Similar ideas could be adapted for marathon runners by including information about current and future training needs.

#### Motivational advice

While it might appear from the outside that runners are a motivated bunch of individuals, getting up at the crack of dawn to hammer out the miles, motivation is always a challenge, especially as training takes its toll on tired bodies or during the dark days of winter for those training for a spring marathon (Masters et al. 1993; Donohue et al. 2006; Krouse et al. 2011; Hammer and Podlog 2016). As such it is interesting to consider how technology might help to motivate runners for their next training session; see (Pilloni et al. 2017). One option is to take advantage of the long history of recommender systems in media, by recommending motivational articles, videos, or podcasts to a runner prior to their training session (Scott et al. 2010). And by incorporating information about the runner’s recent training into the recommendation process, these recommendations can be made even more precise. For example, a new runner might benefit from articles about how to deal with the early base-building stages of marathon training when the miles seem especially long and hard (Han and Xu 2016). A more experienced runner facing into a hard speed-session might benefit from listening to a podcast on the benefits of tempo sessions. Generating an interesting playlist of music and podcasts that is tailored for the duration of a long run might be another useful approach (Álvarez et al. 2019, 2020; Vall et al. 2019; Chen et al. 2020).

#### Active recovery and rehabilitation

Completing a successful cycle of marathon training is about getting the right mix of training, recovery, and rest (Nicolas et al. 2011; Rolf 1995; Bassler 1979). It is all too easy for athletes to focus too much on the training, and in particular the running. Supplementing training runs with active recovery sessions—non-running, exercise sessions designed for recovery—can not only help a runner to recover more effectively but can actually help to make them a better runner. For example, runners who incorporate regular stretching and mobility sessions into their training may do better than those who do not, especially from an injury perspective, but also in terms of race performance.

In the unfortunate event that a runner becomes injured during training, then their rehabilitation will need to not only help them to recover from the injury in question, but also help them to retain their fitness so that they can rejoin their training programme when they recover. All of this requires a careful balance of rehabilitation and recovery effort, for the therapist guiding recovery and the runner anxious to return to normal training. This suggests a role for recommender systems, by supporting the design of a suitable programme of therapy, in response to injury and training needs, and by helping the runner to adhere to this programme; see related work by Fitzgerald et al. (2010), Fitzgerald et al. (2008), Caulfield et al. (2011), O’Huiginn et al. (2009)

#### Exercise and sleep

In addition to their physical training plan and nutrition, marathon runners must pay close attention to their sleep habits. Sleep plays a critical role in recovery from intense exercise, and the quality and duration of sleep have a large effect on the body’s ability to perform at its peak. Many studies have identified the severe effect of even partial sleep deprivation on recovery from intense exercise. For instance, a recent study of cyclists (Rae et al. 2017) looked at the effect of a single night of disturbed sleep on recovery from an intense exercise session. All of the participants experienced a significant reduction in performance and reported feeling sleepier and less motivated to train.

Further research is required to better understand the complex interactions between sleep, recovery and performance (Fullagar et al. 2015), but most runners training for a race understand that good sleep is an essential component of training. This is an area where recommender systems can also make a contribution. Many activity tracking devices also monitor sleep patterns, and using this information to make recommendations for healthier sleep habits is an active research area (Daskalova et al. 2018, 2016; Bauer et al. 2012). There is a clear opportunity here to connect sleep recommendations with richer information about the user’s exercise routine and dietary habits as they prepare for a race.

### On the implications for profiling, personalisation, and recommendation

The aim of this section has been to explore several novel recommendation opportunities that exist in the life of a recreational marathon runner; of course, many of the same opportunities exist for other endurance athletes too. Some of these opportunities should feel familiar to recommender systems researchers, because they bear a strong resemblance to many conventional personalisation and recommendation settings (e.g. recommending routes or partners or media, based on a user’s preferences and interests), but others will be less familiar because they deviate from the preference/ratings-based world of recommendation (e.g. recommending complex training programmes, race planning, fitness estimation).

From a research perspective, these unusual recommendation settings are interesting precisely because they are unusual. Instead of, or in addition to, traditional ratings and preference data, these settings utilise time series data in the form of real-time activities. Such time series can be noisy and unreliable. A runner might stop due to traffic, or forget to start/finish their tracking at the beginning or end of a session, or GPS errors may complicate the extraction of reliable pacing features. At the same time, these data can also provide new types of profiling data. For example, the timing of sessions can provide useful insights into a runner’s preferred schedule: if they usually do their long runs early on a Sunday morning then why not recommend a new video on how to optimise long-runs on Saturday evening? If a runner’s GPS data suggest a proclivity for off-road sessions, then why not recommend the latest trail shoes from their preferred brand, or suggest an upcoming off-road race in their area? If a runner rarely engages in speed sessions during their training, then speed sessions can be suggested and motivated by a relevant blog-post discussing their benefits.

Thus, activity data can serve as a useful source of preferences with which to enrich classical user profiles, to guide personalisation and recommendation. Indeed, this perspective suggests a useful conceptual model of *needs*, *patterns*, and *preferences* for running recommendations^6^. The training needs of a runner, in terms of the fitness requirements of the marathon and a runner’s goal-time expectations, can be tracked by extracting fitness and ability features (e.g. $VO_{2}max$ or *critical speed* from raw activity data over time (see Sect. 4), for example. Periodic training patterns can be identified from the timing, duration, and intensity of activities on a weekly basis and used to estimate important features such as *training load* for use in injury prediction or to generate new training recommendations (see Sects. 5 and 6). And the preferences of runners, such as the types of workouts they prefer or the terrain they usually train on, can provide additional scope for recommending routes and training partners.

## Case studies, research questions and data sources

In the following sections of this paper, we describe four separate case studies to further elaborate on the opportunity for machine learning and recommendation techniques to play a role in the life of a marathon runner. The chosen case studies target several of the primary recommendation tasks outlined in Fig. 1 and as such focus on various aspects of physical training and racing. As mentioned previously, two of the case studies (Sects. 4 and 6) correspond to original work that has not been previously published, while another two case studies (Sects. 5 and 7) summarise previously published work. The decision to include these previously published works as case studies was made in order to provide a more comprehensive account of how recommender systems could play a vital role in many aspects of training.

### Research questions

The following case studies aim to explore a different aspect of marathon training and how it can be supported by the use of recommendation techniques. The following high-level research questions are targeted by these case studies:

1. *RQ 1*—Can we use raw training data to estimate personalised fitness measures during training, without the need for expensive and time-consuming laboratory tests?
2. *RQ 2*—How can we profile a runner’s recent training efforts and use this to recommend modified training sessions as runners adjust their training goals?
3. *RQ 3*—Can we use training disruptions as a proxy for injury and predict the likelihood that a runner will become injured based on their recent training load?
4. *RQ 4*—Can we use prior marathon times to predict challenging but achievable finish-times for an upcoming marathon and recommend a pacing plan to help the runner achieve this time?

### Data sources

All four case studies rely on large-scale datasets which we will summarise here. There are two sources of data used—(1) individual activity sessions from Strava and (2) marathon race records from big-city marathons—each of which is used in different case studies as discussed.

#### The Strava training dataset

Strava is a popular exercise app used by millions of runners and cyclists around the world to track and share their activities. As part of an ongoing data-sharing agreement with the authors, Strava has made available a large subset of anonymised running data for the purpose of research; unfortunately the data sharing agreement does not allow for the sharing or publication of the raw data.

This dataset is used in three of the case studies that follow (Sects. 4–6). In this dataset, each runner *r* is represented as a sequence of time-ordered training activities (Eq. 1).

1 $$\begin{aligned} A(r)= & \big \{A_i(r)\big \}_{i=1}^n \end{aligned}$$

2 $$\begin{aligned} A_i(r)= & \big \{ t_j, d_j\big \}_{j=1}^{m=len(A_i(r))} \end{aligned}$$

3 $$\begin{aligned} paces(A_i(r))= & \big \{t_j/d_j\big \}_{j=1}^m \end{aligned}$$

Each activity $A_i(r)$ is associated with raw distance and timing data, but depending on the device used for tracking, the sampling frequency can vary. In order to normalise and smooth the data, we convert these distances and times into 100m intervals. In other words, each activity is represented as a sequence of time and distance values at 100m intervals as shown in Eq. 2; thus, a 10km activity is made up of 100 timing values corresponding to the time for each 100 m interval. From these we can compute 100m pacing data using Eq. 3. The sex of each runner is also available in this dataset, with age and weight information available for some runners.

#### The marathon race dataset

The final case-study (Sect. 7) uses a marathon dataset collected from the public race records made available from big-city marathons around the world (Smyth and Cunningham 2017b, 2018b; Smyth 2018). Each race record corresponds to an individual runner *r* and a marathon *m* and contains timing information at 5 km intervals and a final finish-time. The dataset also includes the sex of the runner (male or female) and their age group. Since these data are official timing data, it is generally accurate and requires a minimum of processing; interval times are converted into per-kilometre pacing (mins/km) for each 5km interval and the final 2.195 km interval (from the 40 km mark to the finish-line). Thus, each race record is associated with nine separate pacing values for each of the race intervals as in Eq. 4 in addition to sex, age, city, and year information.

4 $$\begin{aligned} c(r, m) = \{p_{5k}, p_{10k}, ..., p_{40k}, p_{42.2k}\} \end{aligned}$$

Although these data are public data, available from marathon web-sites, the authors do not have permission to redistribute it. In the case of the data used in Sect. 7, for the London marathon, it is available from the London marathon website^7^, and an extended list of marathon repositories used to produce this larger marathon race dataset can be found in Smyth (2018).

## Case-study 1—estimating personalised fitness models

Fitness and performance depend on a variety of physiological factors including: how efficiently a runner can consume oxygen (maximum oxygen uptake or $VO_{2}max$) (Noakes 2003; Daniels 2013), the ability of a runner to clear lactate, and other waste products, from their blood during intense exercise^8^ (Billat et al. 2003), and their running economy (Anderson 1996). Determining these factors usually requires runners to perform time-consuming and expensive laboratory evaluations, but in this work we attempt to estimate these factors using the type of raw activity/training data—a time-series of incremental distances, speed, or pace—routinely collected by most fitness apps. If we can infer such fitness indicators from raw training data, then it may provide recreational runners with access to reliable indicators of fitness that would otherwise be out of reach. And such fitness indicators have the potential to play a critical role in other aspects of their training including the fine-tuning of their training programmes, improved models of training load, or better race-day predictions; see also recent work by Emig and Peltonen (2020).

### Mining fitness models from training data

In the Strava dataset, each runner *r* is represented as a set of training activities and each activity corresponds to a time-series of pacing values at 100m intervals (Eqs. 1–3). The key research question here is whether it is possible to infer common fitness models directly from these data and without the need for the type of carefully controlled and supervised *maximal effort* tests (Billat et al. 1996) that are normally required. To do this we need to estimate comparable *maximal effort* paces from raw training data, which requires three steps:

1. First, we convert the distance and time data in each activity into corresponding pacing data (mins/km) as in Eq. 3.
2. Second, for each *activity* we determine the fastest paces over all possible distance intervals, as shown in Eq. 5.
3. Third, for a given activity $A_i(r)$, we compute the cumulative fastest paces, for all possible (100m) distance intervals, so far seen in *r*’s training (for activities $A_1(r),...,A_i(r)$) as in Eq. 6. For example, the cumulative fastest 5k pace associated with $A_i(r)$ is the fastest 5k pace ($m=50$) seen so far in $A_1(r),...,A_i(r)$.

In what follows, we will describe how these paces can be used as the basis for a series of different fitness estimation models by using them to estimate a number of common fitness features. Each model can be used to make a fitness prediction for a given week of training (*w*) based on the fitness features calculated from the pacing data available up to that week.

5 $$\begin{aligned} fastest\big (A_i(r)\big )= & \min _{w=1...m}rolling\Big (paces\big (A_i(r)\big ), w\Big ) \end{aligned}$$

6 $$\begin{aligned} cumfastest\big (A_i(r)\big )= & \min _{k=1...i}fastest\big (A_i(r)\big ) \end{aligned}$$

#### Fastest-pace (FP) model

We can use these cumulative fastest paces directly as an initial baseline fitness model by focusing on certain distances, for which we can expect runners to have engaged in maximal effort sessions during their training. For example, many marathon runners will benchmark their training progress by competing in other (shorter distance) races during training, with 5 km and 10 km races being the most popular distances. Thus, for each runner, and a given week of training *w*, this *fastest-pace* (FP) model uses the three features shown in Table 1.

**Table 1:** The features used in the FP model for a given week of training (*w*); that is, these features are computed based on training up to an including week *w*

	Feature	Description
1	*Fastest-pace-1500 m(w)*	The runner’s fastest 1500 m pace so far up to week *w*
2	*Fastest-pace-5 k(w)*	The runner’s fastest 5 km pace so far up to week *w*
3	*Fastest-pace-10 k(w)*	The runner’s fastest 10 km pace so far up to week *w*

While this is a simple model, it provides a useful benchmark against which to evaluate the more physiologically sophisticated models to come. It is important to note that this model is based on the fastest paces that runners have *happened* to run during training. While some of these may naturally occur as a result of races or programme-prescribed time-trials, it is not necessary for the runner to complete specific (maximal-effort) time trials over prescribed distances.

#### Functional threshold pace (FTP) model

An athlete’s *functional threshold pace* (FTP) is the fastest pace that can be sustained over a 45–60-min period, and this is an important fitness metric used by the popular TrainingPeaks^9^ service. FTP is related to the concept of *lactate threshold* (Billat et al. 2003; Faude et al. 2009; Poole et al. 2008) which is a measure of intensity often used in laboratory-based fitness evaluations. We can calculate a runner’s FTP, at different points in their training programme by computing FTP for specific time durations, so that $FTP\big (A_i(r), t\big )$ is the fastest pace that *r* has run for *t* minutes during training, up to and including activity, $A_i(r)$. We compute four FTP values corresponding to 45-, 50-, 55-, and 60-min durations leading to the four features shown in Table 2.

**Table 2:** The features used in the FTP model for a given week of training (*w*); that is, these features are computed based on training up to an including week *w*

	Feature	Description
1	*Ftp-45(w)*	The runner’s fastest pace for 45 min so far, up to week *w*
2	*Ftp-50(w)*	The runner’s fastest pace for 50 min so far, up to week *w*
3	*Ftp-55(w)*	The runner’s fastest pace for 55 min so far, up to week *w*
4	*Ftp-60(w)*	The runner’s fastest pace for 60 min so far, up to week *w*

In this way the FTP status of a runner during training is represented by a sequence of these FTP values, which can be expected to change (improve) as training progresses, all going well. As with the fastest-pace models, these fastest paces over given times are assumed to have occurred naturally during a runner’s training, although training programmes often include time-trials that will match some or all of the above durations.

#### $VO_{2}max$ model

As mentioned previously, $VO_{2}max$ is another common fitness metric. It measures the maximal rate of oxygen consumption during incremental exercise (Noakes 2003; Daniels 2013; Billat et al. 1994). $VO_{2}max$ is usually determined in a laboratory setting, as part of an incremental treadmill test, using oxygen mask to measure oxygen uptake volume (Billat et al. 1994), but it can also be estimated using a recent maximal effort pace by the Daniels and Gilbert formula (Daniels 2013) shown in Eqs. 7–9; *t* is time in minutes and *v* is velocity in metres per minute.

7 $$\begin{aligned} max(t)&= 0.8 +0.1894393\cdot e^{-0.012778\cdot t}\nonumber \\&+0.2989558\cdot e^{-0.1932605\cdot t} \end{aligned}$$

8 $$\begin{aligned} VO_2(v)= & -4.6 + 0.182258\bullet v \nonumber \\&+ 0.000104\bullet v^2 \end{aligned}$$

9 $$\begin{aligned} VO_2~max(t,v)= & \frac{VO_2(v)}{max(t)} \end{aligned}$$

We can use this to estimate the $VO_{2}max$ of a runner based on their fastest pace (converted to velocity) for distances in the range 1500 m to 30k m (in 100 m intervals), and from these we compute mean and standard deviation values to use as the runner’s current $VO_{2}max$ estimate. In other words, this model uses the two features shown in Table 3.

**Table 3:** The features used in the $VO_{2}max$ model for a given week of training (*w*), based on the fastest paces over distances between 1500 m–30 km; that is, these features are computed based on training up to an including week *w*

	Feature	Description
1	*Mean* $VO_{2}max(w)$	The runner’s mean $VO_{2}max$, so far, up to week *w*
2	*STD* $VO_{2}max(w)$	The runner’s standard deviation of $VO_{2}max$

### Critical velocity (CV) model

The connection between fatigue and exercise performance is linked to the concept of *critical power* (CP) or *critical velocity* (CV), which describes the tolerable duration of intense exercise. In running, the relationship between speed (S) and time to exhaustion, $T_{lim}$, is hyperbolic over different time periods (Muniz-Pumares et al. 2019). The asymptote of the hyperbola is known as *critical speed* (CS) and the curvature constant (D’) represents the finite amount of exercise that can be performed faster than CS. Most athletes can run at their CS for approximately 20–45 min, and CS and D’ have been used as predictors of fitness (Florence and Weir 1997); see also (Emig and Peltonen 2020) for related ideas.

10 $$\begin{aligned} D = D' + CS\cdot T_{lim} \end{aligned}$$

Briefly, CS and D’ can be derived directly from the slope and y-intercept of a linear regression line fit between D and $T_{lim}$ as in Eq. 10. Then, by using the cumulative fastest paces over various distances (1500 m–30 km), at a given point in training, we can estimate $T_{lim}$ for each of these distances. For example, if the fastest pace for a runner over 3 km is 4 min/km, then their $T_{lim}$ for 3 km is 12 min (or 720 s). Obviously this is a $T_{lim}$ estimate only and is all but guaranteed to be an overestimate, since it was likely drawn from an activity where the runner did not run 3 km to exhaustion. Nevertheless, if we utilise our fastest paces in this way, then we can use a linear regression to estimate CS and D’ at different points in training; see also recent work by Smyth and Muniz-Pumares (2020) albeit using a more limited set of distances during the estimation of $T_{lim}$. Thus, for a given week *w*, the CV model uses two features as shown in Table 4.

**Table 4:** The features used in the *CV* model for a given week of training (*w*), based on the fastest paces up to an including week *w*

	Feature	Description
1.	*CS(w)*	The runner’s CS, based on fastest paces up to week *w*.
2.	*D’(w)*	The corresponding D’ value, based on fastest paces up to week *w*.

### Evaluation

We evaluate these four models based on their ability to predict marathon performance, which is a common approach for evaluating other types of fitness estimates (Florence and Weir 1997).

#### Methods

The Strava dataset is used for this evaluation. We select 1,857,698 training (and race) activities logged by 31,221 runners (74% male, 26% female) who competed in Dublin, London, and New York Marathons during the period 2014–2017. The dataset includes all of the training activities associated with these runners for the 16 weeks prior to each race. As mentioned in Sect. 3.2, each activity comprises a list of timing and pacing data at 100m intervals.

For each training week, we compute our four fitness models using the cumulative fastest paces observed up until that point in training. We convert their fitness estimates into weekly representations (one for each of the four fitness models) covering the training period from 11 weeks prior to race-day up until race-day. We focus on this 11 week period because most runners have begun their training in earnest 11 weeks from race-day, and because it also means that we should have reasonably stable fastest-pace estimates by week 11, based on up to the previous 5 weeks of training. Each runner is associated with four separate instance representations—one for each of the fitness models, *FP*, *FTP*, $VO_{2}max$, and *CV*—with each representation comprising a set of weekly fitness features plus a runner’s age, sex, and marathon finish-time. We also produce a fifth representation (*CB*) based on the *combination* of the four separate representations by concatenating their weekly fitness features.

To predict a runner’s marathon time, we test *Bayesian ridge* (BR), *decision trees* (DT), *elastic nets* (EN), *gradient boosting* (GB), *linear regression* (LR), and *random forests* (RF) methods, using the standard SciKitLearn^10^ implementations of these machine learning approaches. For this experiment we did not engage in extensive hyper-parameter tuning and the following default configurations were used: BR with $max~iterations=300$; DT with *mean squared error* used to measure the quality of the splits; EN with $\alpha =1$ and $L1~ratio=0.5$; GB using *least squared regression* loss function, a *learning rate* of 0.1, and 100 boosting stages; RF with $n=100$ estimators and with *mean squared error* used to measure the quality of the splits.

A standard tenfold cross-validation approach is used to evaluate the prediction error: 10% of training instances are used as a test set with their fitness features (plus age and sex) used to predict marathon time, using a model trained on the remaining 90% of instances, and being careful to ensure that the test runner has none of their own instances in the training set. Then, we compare the predicted finish-time to the actual marathon time of the test runner to calculate the *prediction error* from the mean absolute difference between the predicted and actual finish-times; these errors are averaged across the tenfolds for each algorithm and representation.

#### Results

Table 5 shows the mean prediction error for each combination of representation and algorithm, averaged over all training weeks. Briefly, the combined *CB* representation offers the lowest prediction error ($\mu _R=16.19$ mins), averaged across individual algorithms, while the GB algorithm offers the lowest prediction error ($\mu _A=15.17$ mins), averaged for representations. The differences between all of these mean prediction errors are statistically significant ($p<0.01$) based on a one-tailed ANOVA and Tukey's range test. Notably, the best prediction error for any given representation is always associated with the GB algorithm; once again these individual differences in prediction error for GB are all statistically significant at $p<0.01$, in comparison with all other combinations of algorithm and representation.

**Table 5:** The average absolute prediction error (in minutes) based on prediction algorithm (rows) and fitness model representations (columns)

	CB	CV	FP	FTP	VO2	$\mu _A$
BR	15.24	21.01	16.20	16.83	30.97	*20.05*
DT	20.92	21.70	23.01	23.32	21.14	*22.02*
EN	16.06	25.11	17.39	17.59	31.88	*21.60*
GB	14.34	14.89	15.79	16.17	14.65	*15.17*
LR	15.24	21.01	16.20	16.83	30.97	*20.05*
RF	15.35	16.23	17.14	17.20	15.86	*16.35*
$\mu _R$	*16.19*	*19.99*	*17.62*	*17.99*	*24.24*	*19.21*

The prediction algorithms are: Bayesian ridge (BR), decision trees (DT), elastic nets (EN), gradient boosting (GB), linear regression (LR), and random forests (RF). The fitness model representations are: the combined representation (CB), the critical velocity model (CV), the fastest pace model (FP), the functional threshold pace model (FTP), and the $VO_{2}max$ model (VO2). The mean prediction error by algorithm and representation are shown as $\mu _A$ and $\mu _R$, respectively

The italics indicate the means of the errors when aggregated by representation or algorithm

The single best performing predictor (GB-CB) is capable of estimating marathon times that are within 14.34 mins ($<6\%$) of actual race-times. As a baseline reference, this compares favourably with state-of-the-art marathon predictors (Keogh et al. 2019), which are associated with an average error of 14.35 mins, based on a set of 19 different prediction formulas, many of which require costly, laboratory-based measures of fitness and ability. That our approach achieved similar prediction performance without the need for laboratory testing speaks to the potential of the proposed approach, and it provides a significant benefit for recreational runners by using their raw training data without the need for laboratory controlled testing. As another useful benchmark, in a related study by Berndsen et al. (2017), the average error for the classic Riegel race prediction formula (Riegel 1981), which is often used by recreational runners, was approximately 10% (>23 min) across a similar range of finish-times to those used here.

**Fig. 2:**
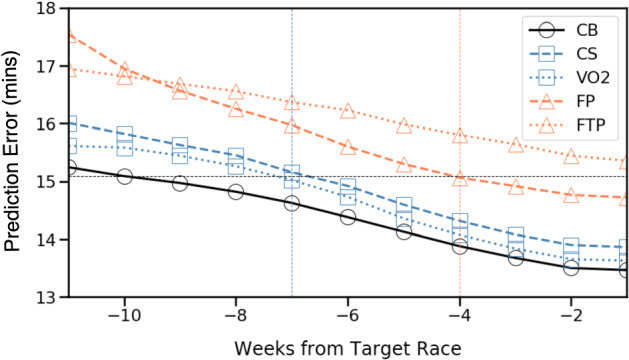
The absolute prediction error (minutes) by weeks before race-day using the GB algorithm and for different fitness models, including the combined (CB) fitness model

In Fig. 2 we plot prediction error by week of training using GB with each of the fitness representations. Errors steadily reduce as training progresses, suggesting more and more accurate estimates of runner fitness, at least in terms of their ability to complete the marathon. Note how 10 weeks before the race, the GB-CB model is able to predict marathon finish-times with an error of about 15 minutes. CV and $VO_{2}max$ achieve this level of accuracy 3 weeks later. The FP representation achieves it another 3 weeks after that while the FTP representation never achieves this accuracy level at all. These differences are important. The benefit of an accurate early fitness estimate is that it provides the basis for a more optimal training plan that is tuned to the ability of an individual runner. It helps a runner to determine how they should train, and early and accurate fitness estimates are key to this, so that there is enough time to take advantage of this training. In other words, the ability to produce an accurate fitness model early in training can provide an important foundation for a whole host of future recommendations, from the fine-tuning of training to goal-time prediction and pace planning.

### Discussion and limitations

This work demonstrates how raw activity data can be used to directly estimate several common fitness models. The resulting models offer reasonably accurate estimates of fitness, even though there are no guarantees that runners in our data have performed suitable time trials or similar maximal effort tests during normal training. Of course, it will be important to conduct live-user trials to compare these estimates to laboratory measures and such a live-subject trial is currently underway.

In relation to the race-time predictions, it is worth noting that more accurate predictions can be achieved by incorporating a greater range of features during prediction; the aim in this case-study was to provide evidence to support the effectiveness of fitness model prediction more than it was to develop a best-in-class race-time prediction tool. For example, recent work by Smyth and Cunningham (2018a) and Emig and Peltonen (2020) has demonstrated superior race-prediction error rates in the 2-3% range, while incorporating training features into a similar approach reduced error rates to approximately 5% (Berndsen et al. 2020d). The point is that the ability to estimate physiological models of fitness can be expected to drive such prediction improvements, in concert with other predictive features.

An important limitation is that these techniques rely on pacing data only—and relatively coarse-grained (100m) pacing data at that—and there is further room for improvement by leveraging additional types of activity data, such as heart-rate data, with the potential to significantly improve the accuracy of models such as $VO_{2}max$ and CV in particular.

## Case-study 2—recommending personalised training sessions

One of the most challenging aspects of training for a marathon, especially for novices, is knowing *how* to train: knowing how often to run, how far to run, and how fast to run. Training programmes usually involve a complicated mix of short/long and slow/fast sessions and most runners will select one based on their goal-time. While it would be wrong to view these as unsophisticated training plans—after all they are usually designed by experienced coaches and as such bring a wealth of coaching experience and a knowledge of human physiology to the task—they are nevertheless fairly blunt instruments because they typically target a wide range of finish-times (e.g. 4–5 h) and are rarely tailored to the individual runner. It is not surprising then, that the idea of a personalised training plan, one that is tailored to a specific runner and that adapts to their training progress, has long been an ideal for many marathoners. But without a coach, such personalised plans are likely out of reach for most runners. And automatically generating such plans is far from trivial, because it requires a deep domain model combining human physiology with knowledge of the particular requirements of the marathon distance.

We discuss one attempt at generating personalised training plans in this case-study, by reusing the training of similar runners. Unlike the previous case-study, which represents original, as yet unpublished research, this case-study summarises recently published work (Feely et al. 2020b, a), which is included here because it adds another dimension to our vision of how recommender systems can be used to support marathon runners.

### A case-based reasoning approach to training recommendation

Case-based reasoning solves new problems by retrieving and reusing the solutions to similar problems that occurred in the past, and CBR approaches have proven to be especially useful in domains, and for tasks, which lack a strong, complete domain model, but where an abundance of past cases can be found. This is true for marathon running. The recorded activities of runners, aggregated by week or month, constitute an abundance of training cases, which can be reused to support the training of similar runners in the future.

#### From training sessions to training cases

To test this hypothesis, we have developed a CBR system for finish-time prediction and training recommendation based on cases composed of a runner’s weekly training activities and using the key features shown in Table 6

**Table 6:** The features used in training cases for the prediction of marathon time and for recommending tailored training plans

	Feature	Description
1	*Sex*	The runner’s sex (male or female)
2	*Num sessions*	The *number of sessions* in the current week
3	*Total distance*	*Total distance* in kms for the current week
4	*Mean pace*	*Mean pace* for the week in mins/km
5	*Longest run distance*	*Longest run distance in the current week*
6-8	*Fastest 1 km/5 km/10 km*	Fastest 1/5/10 k paces for the current week
9-11	*Slowest 1 km/5 km/10 km*	Slowest 1/5/10 k paces for the current week
12	*Longest run distance to date*	*Longest distance so far in training*
13-15	*Fastest 1 km/5 km/10 km to date*	Fastest 1/5/10 k paces so far
16-18	*Slowest 1 km/5 km/10 km to date*	Slowest 1/5/10 k paces so far

**Fig. 3:**
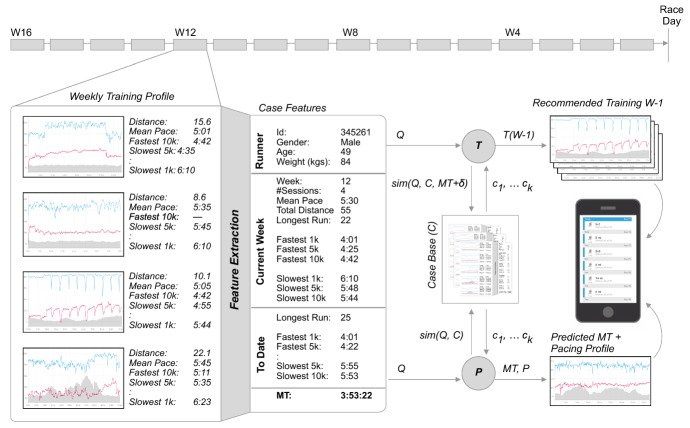
An overview of a case-based reasoning system for supporting marathoners during their training by predicting (P) their estimated marathon time and by recommending (R) a tailored training plan for an adjusted marathon time. Note that although a runner’s age and weight are shown in this figure, these features are not used in the results presented here as they did not substantially improve on prediction accuracy

These features were used because they are commonly associated with aspects of marathon training (Doherty et al. 2019). For example, the duration of *long-runs* is often cited as an important success criteria, while long-distance pacing typically correlates with marathon times. In addition to these features, which represent the current week of training, we also calculate the corresponding features for the training period up to and including the current week (e.g. *longest run distance* to date). Thus, for each runner *r*, we can generate a feature-based description for training week *w*, *F*(*r*, *w*) as shown in Fig. 3 for a runner in week 12 of their training.

Next, we generate a case, *C*(*r*, *w*), representing *r*’s training during week *w*, by associating *F*(*r*, *w*) with their marathon time, *MT*(*r*), and also a pointer to their next week of training, $C(r, w-1)$; see Eq. 11. These cases can be used in two ways: (a) to predict a runner’s marathon time at week *w*, using the *MT* components of similar cases; and (b) to recommend next week’s training schedule, using the $C(r, w-1)$ component of similar cases for a revised goal-time ($MT\pm \delta$).

11 $$\begin{aligned} C(r, w) = \big \{F(r, w), MT(r), C(r, w-1)\big \} \end{aligned}$$

#### Generating training recommendations

In this case-study, we focus on the recommendation task (R) rather than the prediction (P) task; see (Feely et al. 2020b) for further information. Consider a runner *r* in week *w* of a training programme targeting a finish-time of *t* minutes. If *r* wishes to continue to target this finish-time, then they can continue to follow their existing training programme, but what if they decide to target a more ambitious (or less ambitious) time, $t\pm \delta$? How should *r* adjust their training for the coming week(s)? Our solution is to use their training case for week *w*, *C*(*r*, *w*), as a query into a case base of other cases for week *w*, to retrieve the most similar case for another runner who achieved a $t\pm \delta$ finish-time in their target marathon.

To do this we identify a subset of week *w* cases (based on runner sex) with finish-times within 1 minute of $t\pm \delta$, and from these cases we select the single case that is most similar to *C*(*r*, *w*) using a standard Euclidean distance metric, based on the normalised features of the query and these candidate cases. In this way, our target runner *r* will be recommended a new week of training from the training of a similar runner who achieved r’s modified goal-time.

It is worth noting that we select the *single* most similar case because we wish to use its training sessions for week $w-1$ (the next week of training). It is reasonable to consider selecting more than one case, such as the *k* most similar cases. The complication with this is that it introduces the additional problem of how to combine different weeks of training prior to recommendation. The training weeks of the top *k* cases may suggest different numbers of sessions on different days of the week, which will complicate the combination process. In the future it will be important to explore this further, but for now we focus on the single most similar case.

#### From single-week to multi-week cases

So far the focus has been on matching weekly training cases based on a single (current) week of training, but since marathon programmes are typically designed around 4-week training blocks, it is also worth considering a longer, 4-week training period during recommendation. To do this we use an ensemble approach to generate recommendations based on each of the 4 most recent weeks of training. Thus, for example, for week $w=10$, we identify 4 similar cases using the case bases for weeks 10, 11, 12, and 13, and the final recommendation is based on the most similar of these cases.

One problem with this approach is that training plans are not always in sync, because training volumes ramp up and down during each training block. To deal with this, we implement a variation of this 4-week ensemble by *ordering* the 4 training weeks in ascending order of training-load. For example, for week $w=10$, we use cases from weeks 10, 11, 12, 13 ordered by their longest run distance. So the $w-3$ case base contains the training week with the shortest long-run, the $w-2$ case base contains the next shortest long-run, etc. The advantage of this is that it facilitates a better alignment between the training weeks of runners over a 4-week period and in the work of Feely et al. (2020b) this has proven to be a more effective approach than either the single-week approach or the unordered 4-week ensemble.

### Evaluation

Properly evaluating training plan recommendations requires a live-user trial in which runners at least provide their opinions of the recommended training plans, if not avail of them as part of their training so that we can test the eventual race outcome. While this is planned for the future, it is beyond the scope of the present work. In the alternative, we present a more conventional off-line evaluation, using the training data from real runners, to demonstrate the reasonableness of these training plan recommendations.

The main Strava dataset was used, but this time focusing on a subset of 5000 female runners who completed their marathon in 3–5 h and 15,000 male runners who completed their marathons in up to 5 hours. We use a standard tenfold cross-validation to generate training recommendations for each runner *r*, training week *w* and for $-20\le \delta \le 20$ minutes, and we evaluate the recommended training weeks by comparing their total weekly training volume and average weekly pace to the target runner’s volume and pace for their default next week of training ($\delta =0$). We should expect the recommendations generated for faster target finish-times ($\delta <0$) to have a greater training volume and a faster average pace, and vice versa for slower ($\delta >0$) target finish-times.

#### Results

**Fig. 4:**
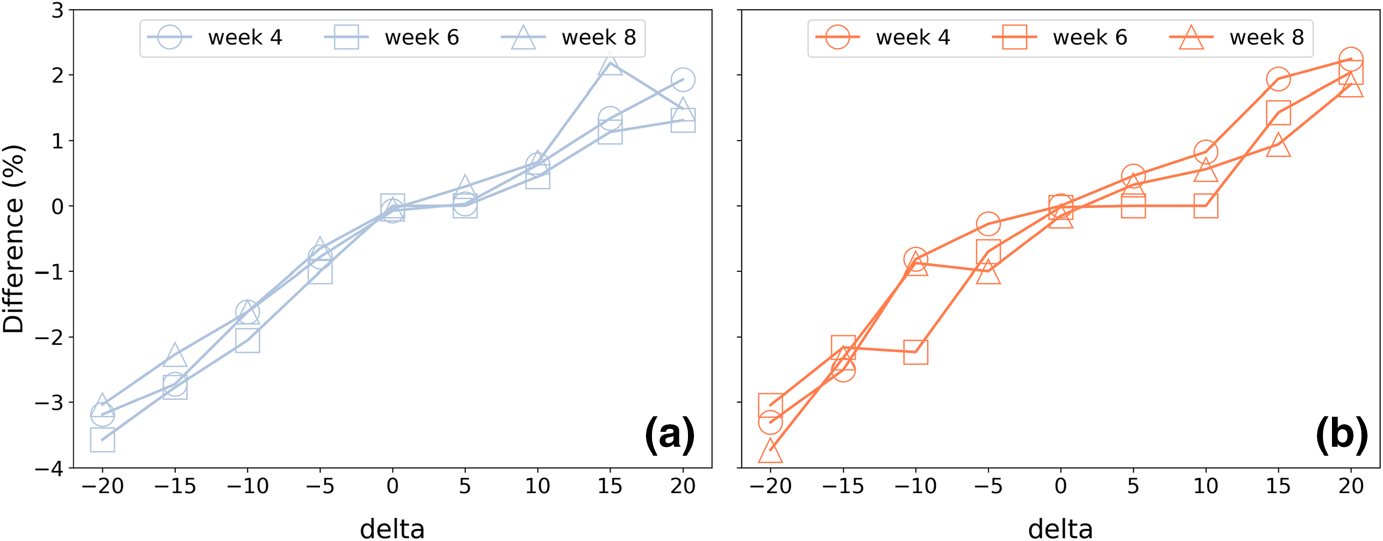
The difference in mean weekly pace (mins/km) for training plans based on adjusted goal-times for **a** men and **b** women during weeks 4, 6, and 8 of training. Note $\delta<$0 implies a goal-time that is delta minutes *faster* than the runner’s current predicted time while a $\delta >0$ indicates a slower *pace*

**Fig. 5:**
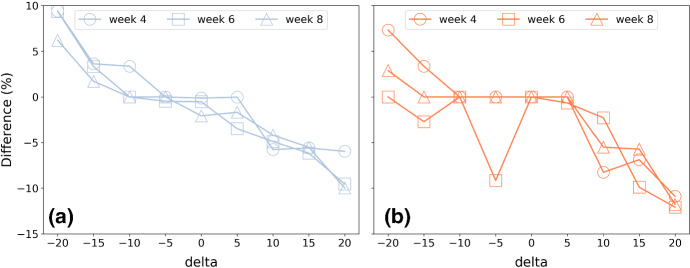
The difference in mean weekly distance (km) for training plans based on adjusted goal-times for **a** men and **b** women during weeks 4, 6, and 8 of training. Note delta$<0$ implies a goal-time that is delta minutes *faster* than the runner’s current predicted time

The results of the experiment are summarised in Figs. 4 and 5 for recommendations generated at weeks 4, 6 and 8 of training, using the ordered 4-week approach described above; additional results are presented in Feely et al. (2020b). In general, the results are consistent with expectations. When runners request training plans that are faster than their current predicted finish-time ($\delta <0$), then mean weekly pace tends to speed-up (a negative % difference as in Fig. 4), while total weekly distance tends to increase (a positive % difference as in Fig. 5). The reverse is true when they request a plan for a slower marathon time.

The changes in pace exhibit a very strong correlation with $\delta$ ($R^2>0.92$ for men and women). The changes in weekly distance are also strongly correlated with $\delta$ for men ($R^2>0.90$), but less so for women ($R^2>0.66$ on average). The relative changes in distance tend to be greater (for a given $\delta$) than the corresponding changes in pace.

### Discussion and limitations

While not definitive, these results are encouraging, especially given the challenges inherent in this particular recommendation task; conventional recommendation techniques have largely focused on recommending simple, atomic items (books, music, movies) rather than complex items, such as training plans, which are made up of a complex mix of interacting elements. The fact that we can generate training plan recommendations that are at least consistent with a runner’s modified goals (in terms of training volume and pace) is an encouraging starting point. And since these plans are based on the real training plans of similar runners, this increases the chances that they will be acceptable to runners.

That being said, it is important to consider the additional risk associated with this form of recommendation if, for example, the most similar runner (used as the basis for next week’s training) is not following a suitable marathon programme. Then, the recommended training plan may not be suitable for the target runner and may do damage to their training. This is not explicitly accounted for in this case-study and, indeed, may be exacerbated by the focus on a single most similar case; reusing and combining the training plans of *k* similar runners may help in this regard. In related work by Berndsen et al. this issue is addressed more directly (Berndsen et al. 2020c) by accounting for training periodisation, for example, and by nudging users towards training behaviours that are more similar to expertly designed plans.

Certainly a more thorough evaluation is needed to build on this initial work. It remains to be seen, for example, how receptive runners are to these recommendations during their own training. How can such recommendations be justified and explained? How should the risks be communicated? Is there evidence that by adapting their training as per the recommendations that they tend to achieve a better outcome? These issues require live-user studies over extended periods of training and this remains an important objective for future work.

## Case-study 3—estimating injury risk during training

As training progresses most runners will begin to feel the burden of week after week of longer, faster, more intense sessions. Most training plans are designed to gradually increase training load in a way that is safe for most runners but great care needs to be taken to avoid over-training and the injury risk that this entails. This does not always work out and so-called running-related injuries (RRIs) are one of the most common reasons why people fail to make it to the start-line (Clough et al. 1987).

In this original case-study we use the Strava activity dataset from Sect. 4 (1,857,698 activities for 31,122 runners of the Dublin, London, and New York marathons during the 2014-2017 period) to estimate the risk of a runner developing a RRI, so that we can alert runners if they are at a greater risk than expected. If they are at greater risk, then runners can choose to temporarily reduce their training load or intensity. Alternatively, they can supplement their training with more active recovery (stretching, strength and conditioning, better sleep, etc.).

In general, injury prediction is a challenging task (Carey et al. 2017; Kampakis 2016; Rossi et al. 2018) but it is especially demanding here for at least two reasons. First and foremost, we do not have any information about a runner’s injury history or status, which can be an important factor when predicting future injuries (Hulme et al. 2017; van der Worp et al. 2015; Saragiotto et al. 2014). The second problem is that the activity dataset does not include explicit injury data, so we cannot even tell if the runner becomes injured during their training. Instead, we use disruptions in a runner’s training activities—extended breaks in training—as a proxy for likely injuries.

In the following, we describe how we identify and use these training disruptions to predict future injuries and to estimate injury risk, using the Strava dataset. Note that we do not attempt to distinguish between different types of injuries—soft-tissue, overuse, accidental, etc.—mainly because the data do not support it.

### Using training disruption as a proxy for injury

For some training activity $A_{i}(r)$ we use $next(A_{i}(r))$ to denote the number of days between activity $A_{i}(r)$ and the next activity, $A_{i+1}(r)$ as shown in Eq. 12; in what follows we may refer to $A_{i}(r)$ as $A_{i}$ where the correspondence to runner *r* is unambiguous.

12 $$\begin{aligned} next\big (A_{i}(r)\big ) = A_{i+1}.d - A_{i}.d \end{aligned}$$

While short breaks of up to a week or so might occur for reasons other than injury (busy at work, travelling, illness, lack of motivation, etc.) longer breaks of more than 10–14 days are likely to be more reliable indicators of some injury-related issue that is preventing the runner from training. Thus, we are interested in identifying sections of a runner’s training where the next training activity is *more* than *n* days in the future, for $n=7, 10, 14$, as in Eq. 13; $break\big (A_{i}(r), m, n\big )$ is true, if and only if there exists some future training activity $A_{j}(r)$, *within* *m* days of $A_{i}(r)$, such that $A_{j}(r)$ represents the beginning of a break of *more* than *n* consecutive days.

13 $$\begin{aligned} break\big (A_{i}(r), n, m\big ) \iff \exists A_{j}:i<j<i+m \wedge next\big (A_{j}\big )>n \end{aligned}$$

### Representing activity features

Next we define the features that will be used to predict these disruptions. We propose four groups of features as follows:

#### Baseline features (B)

Baseline features (Table 7) include the runner’s *age* (in years), their *sex* (male or female), and *days-from-race*, the number of days that the current activity is from the target race; previous studies have reported varying relationships between RRIs and age and gender (Agresta et al. 2018; Napier et al. 2018).

**Table 7:** The baseline features (B) used during injury prediction and risk assessment

	Feature	Description
1	*Sex*	The runner’s sex (male or female)
2	*Age*	The runner’s age in years
3	*Days-before-race*	The number of days until the target race

#### Ability features (A)

As runners train, their performance over a range of distances should improve, but the rate of these improvements may signal over/under training, which will likely influence injury risk. Our instances include three features based on the fastest pace observed for a runner over 1 km, 5 km, and 10 km distances, as shown in Table 8.

**Table 8:** The ability features (A), based on fastest paces during training, used during injury prediction and risk assessment

	Feature	Description
1	*Fastest-pace-1 k(d)*	Runner’s fastest 1 k pace up to *d* days before race-day
2	*Fastest-pace-5 k(d)*	Runner’s fastest 5 k pace up to *d* days before race-day
3	*Fastest-pace-10 k(d)*	Runner’s fastest 10 k pace up to *d* days before race-day

These are similar to the fastest-pace features used in Sect. 4, and these particular distances have been chosen because they represent likely time-trial distances for runner during training: many training programmes will include 5k and 10k races, for example. These features contain no explicit information about any training disruptions but they are included on the grounds that the ability of a runner may help to predict their risk of disrupted training; for example, faster, more able runners are likely more experienced and more diligent in their training, and as such they may experience a lower likelihood of a training disruption.

#### Disruption history features (H)

Whether or not a runner has already experienced a training disruption, may predict further disruptions in the future; for example, (Hulme et al. 2017; van der Worp et al. 2015; Saragiotto et al. 2014) conclude that a history of running-related injury is a risk factor for future injuries. Hence, we include two features related to past disruptions as shown in Table 9.

**Table 9:** The disruption history features (H) capture whether a runner had a previous disruption up to *d* days before race-day and the number of days since such a disruption if one occurred

	Feature	Description
1	*Has-prev-break (d)*	True if a runner had a break up to *d* days before race-day
2	*Days-since-break (d)*	Number of days since the most recent target break

#### Training load features (T)

Training load is believed to be an especially significant factor when it comes to whether a runner is likely to become injured (Thornton et al. 2017; Malisoux et al. 2015). We measure training load using the *acute-chronic ratio* (ACR) metric (Barros et al. 2017; Bowen et al. 2019). ACR is usually defined as a runner’s current weekly training load (*acute* load) divided by the 4-week rolling average of their weekly load (*chronic* load). Thus, an $ACR>1$ means that their current week has a higher load than their 4-week average. We use weekly training volume (total distance) as a basic estimate of training load, leaving more sophisticated measures for future work. For this study we calculate three ACR features to reflect different chronic-load periods as shown in Table 10.

**Table 10:** The training load features (T) estimate different training load ratios based on the ratio between the current week’s training load and the average training load of the previous 4, 6, or 8 weeks

	Feature	Description
1	*Acr-1w-4w(d)*	The current week’s load divided by the mean of the last 4 weeks
2	*Acr-1w-6w(d)*	The current week’s load divided by the mean of the last 6 weeks
3	*Acr-1w-8w(d)*	The current week’s load divided by the mean of the last 8 weeks

By considering these variations on how the chronic training load is calculated, we can obtain a more detailed picture of how a runner’s training load has evolved over an extended period of time. Also, recent work has speculated about the need to consider changes in training load over extended periods of time (Damsted et al. 2018) when it comes to injury prediction.

### Evaluation

For evaluation purposes, we frame our approach as an imbalanced binary classification task (Saito and Rehmsmeier 2015)—given some target activity $A_{i}(r)$ the task is to predict whether the runner will experience a training disruption of $>n$ days at some point in the next *m* days, for different values of *n* and *m*.

#### Methods

We evaluate performance by using 3 representative machine learning algorithms (standard Scikit-learn implementations)—logistic regression (LR, using an *L2* norm for penalisation, with a stopping tolerance of 0.0001, and a regularisation strength of 1), random forests (RF, with 100 estimators), and Gaussian Naive Bayes (NB)—to predict breaks of varying durations ($n=7, 10, 14~days$) and for different look-ahead periods ($m=7, 14, 21, 28, \infty$ days^11^), using the B, A, H, T feature sets, and for different training dataset sizes (50k, 100k, 500k, all 1.8m Strava instances).

For each configuration we run a tenfold cross-validation to train and test models from the data using each algorithm; for example, in one configuration, we train and test a random forest model to predict training disruptions of $>14$ days up to race-day ($m=\infty$) using training datasets of 100k observations.

Since this work is an example of an imbalanced machine learning problem, with positive instances—those indicating the presence of a training disruption—in the minority, we use random undersampling (He and Garcia 2009; Chawla 2010) to re-balance the number of positive and negative training instances, by deleting negative instances at random; in line with best practice resampling is performed after splitting training and test data during each cross-validation fold.

In this evaluation, we consider two classification outcomes: (1) the accuracy of the predicted class, to determine how often we can correctly predict whether a runner will succumb to injury in the future; and (2) the probability of the positive class as a type of *injury risk score*, which is closely related to the type of *lift analysis* that is often used to evaluate the efficacy of churn prediction models (Hung et al. 2006) or marketing response rates (Piatetsky-Shapiro and Masand 1999).

#### Results

**Fig. 6:**
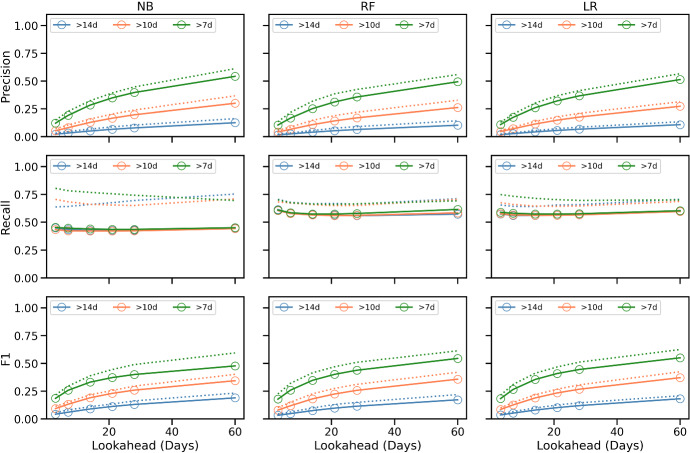
The precision, recall, and F1 results for classifiers (NB, RF, LR), averaged overall all dataset sizes (50 k, 100 k, 500 k, 1.8 m), for different break-types ($>7d$, $>10d$, $>14d$), and by look-ahead days (7*d*, 14*d*, 21*d*, 28*d*, and $\infty$, which means up until race-day)

Figure 6 shows the classification accuracy results for each algorithm, break-type (*n*), and look-ahead (*m*), using three common accuracy metrics, *precision*, *recall*, and *F1*. It is worth noting that in this type of imbalanced classification task, where there are many negative instances, precision is widely accepted as the most suitable evaluation metric (Davis and Goadrich 2006; Saito and Rehmsmeier 2015). Precision evaluates the fraction of positive predictions that are correct—essentially estimating the probability of correctly classifying training disruptions of a given duration—which is not effected by the number of negative examples. For completeness, we also show the recall and F1 results.

In each graph, the solid lines represent the average scores (whether precision, recall, or F1), computed over all of the feature sets in our evaluation, whereas the dotted lines show the corresponding scores for the single best model in each trial; that is, for a given algorithm, break-type, and look-ahead. For example, for RF we can see how the precision of predicting short ($>7d$) training disruptions up to 60 days from race-day, is just under 0.5, on average over all models, but among these models the best precision we obtain is just over 0.5, or about 10% better than the mean.

These results suggest that the classifiers are not performing well enough to be useful in practice: precision rarely exceeds 0.5 and when it comes to predicting longer training disruptions ($>14d$)—those most likely to be associated with injury—then precision typically falls below 0.1 regardless of look-ahead. In other words, when the classifier predicts that a runner will experience a $>14$-day training disruption, then it is correct less than 10% of the time. This is not accurate enough to be useful to runners and, although precision improves for shorter disruption durations, these are less likely to be reliably associated with genuine injuries. Disappointing as these results are, they are not so surprising given the limited information that is available in the activity dataset.

**Fig. 7:**
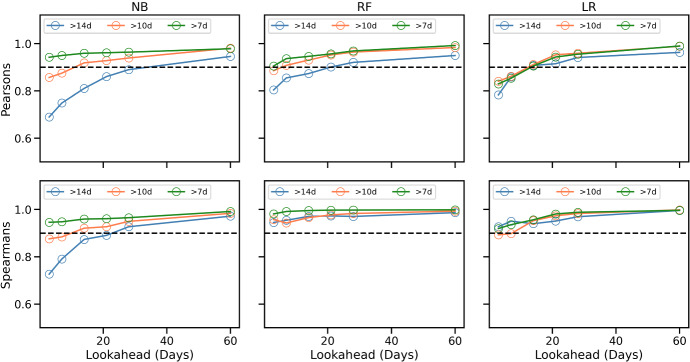
The (max) correlation coefficients (Pearsons and Spearmans) between the positive class probability scores and the frequency of break-types among runners with these scores, for the different algorithms and break-types

Nevertheless, these results do not necessarily mean that there is no value in the approach. For example, when we use the probability of the positive class as a type of *injury risk score* we find a strong correlation between the positive class probabilities and the incidence of training disruptions in test data, particularly for the Spearman rank correlation coefficient and RF, which always enjoys correlation values in excess of 0.9; the Spearman values tend to be higher than Pearson because the relationship between the positive probabilities and the incidence of training disruptions tends to be nonlinear. This is evident in Fig. 7, which shows the correlation results for each look-ahead value, using the full complement of data (1.8m instances) for each algorithm and break-type.

### Discussion and limitations

In this case study, the aim was to support runners during their training by alerting them if their training patterns suggest a higher risk of injury. Ideally we wanted to be able to reliably predict whether a given training pattern was likely to lead to a future injury, but the results presented did not support this. However, the positive (injury) class probability score, produced as a side-effect of classification, proved to be strongly correlated with the likelihood of future injury and, as such, may provide a reliable measure of injury risk. The benefit of this is that it makes it possible to inform runners about their current level of injury risk, using alerts or notifications such as the following or other visual indicators:

*Alert: your current injury risk score is 0.8. At least 40% of runners with a similar risk score go on to experience a training disruption of* >14 *days before race-day.*

*Alert: your current injury risk score is 0.8. This means you are* $>3$*-times more likely to suffer from a training disruption of* $>14$ *days than a typical runner.*

One limitation of this work is that we are using training disruptions as a proxy for injuries, because our dataset does not include any explicit injury data and it is, as yet, unclear whether (long) training disruptions can be used as reliable proxies for injury. Another limitation relates to the features used for prediction. Certainly it may be feasible to consider additional features to model the onset and recovery from previous disruptions, either as a way to more reliably identify true injury breaks, or as a way to infer how a runner has recovered from previous injuries. For example, if a runner is becoming injured then we might expect their training patterns to change before the training break. If they return to training too quickly, then we might find that this is a stronger predictor of future injuries. Certainly these ideas are worthy of future consideration and may help to improve the results.

Another methodological limitation with the approach taken here is that it fails to properly account for runners who become injured so close to the marathon that they fail to participate in the race: such runners are not present in our dataset. This means that we cannot properly evaluate this approach when it comes to injuries that occur in the 1-3 weeks before a race, which are more likely to see a runner dropping out; the approach may work for these runners but our evaluation cannot test it. Indeed there may also be issues related to class skew if the frequency or nature of disruptions depends on the prediction horizon (Boyd et al. 2012). It is difficult to analyse this further, given that the current dataset is likely to be missing runners who become injured late in training, further highlighting the need to address this shortcoming in the current dataset. As part of future work we plan to explore whether it may be possible to identify runners who appear to be training for a particular marathon but who fail to participate due to a late training disruption. One way to identify these runners might be by clustering runners based on their similarity to runners who are known to be training for a given marathon.

Finally, future work will explore more sophisticated estimates of training load, for example by using heart-rate data, when available, to determine effort and intensity. That being said, it is encouraging that even though we are using simple, volume-based, estimates of training load, we can still produce a useful estimate of injury risk; it is also worth pointing out that while ACR proved to be a useful measure of training load in this instance, its general utility remains a matter of debate (Bornn et al. 2019).

## Case-study 4—personal-best prediction and pacing recommendation

In choosing case studies for this paper we have followed the chronology of a typical marathon runner during training: we started by estimating their early and evolving fitness in Sect. 4; then, we attempted to personalise their training as their race-goals evolved in Sect. 5; next, we monitored their training load to alert them to injury risks in Sect. 6. The central aim has been to get them safely to race-day. Now it is time to prepare them for their race and help them to secure a finish-time to be proud of and that does justice to their training efforts.

Race planning is an especially important task when it comes to competing in an endurance event such as the marathon, because there is plenty of time for things to go wrong. It is rarely a good idea to turn up at the start-line without a pacing plan. Instead, marathon runners are advised to estimate their likely finish-time and work out their pacing for the different phases of the race. Some runners use a pacing plan for each km/mile of the race. For others, having a pacing target every 5kms or so is sufficient. Either way it is important for runners to calibrate their pacing with respect to realistic finish-time expectations. Otherwise, they run the risk of starting too fast and burning up before the end of the race (Smyth 2018) or even hitting the wall (Stevinson and Biddle 1998; Buman et al. 2009, 2008; Berndsen et al. 2020a). On the other hand, without a plan they may run too conservatively, and cross the finish-line without feeling they have achieved their best time, leaving them disappointed and demoralised.

In practice, different runners will adopt different types of pacing plans. Some common approaches include:

1. *Positive Splits*—runners run the first half of their race faster than the second half;
2. *Negative Splits*—runners run the second half of their race faster than the first half;
3. *Even Splits*—runners run the first half of their race in a similar time to their second half.

Generally speaking, positive splits are much more common among recreational runners—after all, it is only natural for runners to slow during the second half of the race—but more experienced runners often aim for even splits or sometimes negative splits. Obviously pacing will depend on course topology too, and other factors (temperature, wind, etc.), but all other things being equal, even splits are generally viewed as preferable to more extreme positive or negative splits. Certainly, large negative and positive splits tend to be associated with sub-optimal finish-times. In this final case-study we summarise recent work on predicting personal best (PB) finish-times, and recommending pacing plans to help runners achieve these times (Smyth and Cunningham 2017b, 2018a, b), using ideas from case-based reasoning.

### Using CBR to predict PBs and recommend pacing plans

**Fig. 8:**
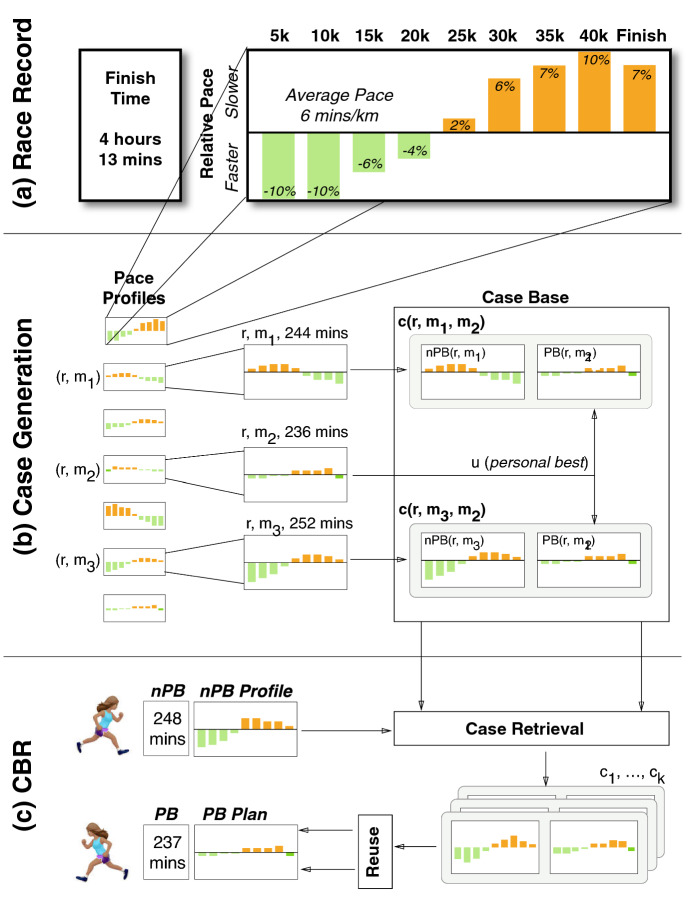
Races, cases, predictions, and recommendations. **a** An example race record for a runner, showing a finish-time and a pacing profile containing pacing data for each of the 5 km race segments. **b** Converting race records into cases by pairing a runner’s non-PB races with their fastest (PB) race. **c** An overview of the CBR prediction and recommendation process: given an nPB race record as a query, the system retrieves a set of *k* cases with *similar* nPB races, and combines these to generate a personal best finish-time prediction and a recommended pacing plan to achieve this finish-time

Instead of using training activities, in this case-study we use past marathons race records as the basis for prediction and recommendation; see Sect. 3.2.2. Each marathon is represented at a set of paces (mins/km) at 5km intervals plus the pace for the final 2.2 km as in Eq. 4 and Fig. 8a; note that in Fig. 8a we convert actual segment paces into relative paces calculated with respect to a runner’s mean race-pace. Then, for each runner with at least two races, we can identify their fastest marathon as their PB, and for we generate a set of cases by pairing each of their non-PB races with their single PB race as in Eq. 14. For example, in Fig. 8b we see a runner, *r*, who has completed three marathons ($m_1, m_2, m_3$) with $m_2$ as their PB, leading to two cases, $c(r, m_1, m_2)$ and $c(r, m_3, m_2)$.

14 $$\begin{aligned} c_{ij}(r, m_i, m_j)=\Big \langle nPB_i(r, m_i), PB(r, m_j) \Big \rangle \end{aligned}$$

Thus, each case represents the progression of a runner, from some non-PB marathon finish-time to a PB finish-time, encoding the different pacing profiles of the non-PB and PB races. Then, given a new target runner, trying to achieve a new PB, and with some prior non-PB marathon, we can use their non-PB as a query into the case base to retrieve the *k* most similar cases—we use a simple cosine similarity metric—based on their non-PB pacing profiles and finish-times; we also separate the cases for male and female runners as performance and pacing differs between the sexes. The resulting *k* cases have similar non-PB races to the target runner and, as such, their subsequent PBs may be informative with respect to the target runner’s PB prospects.

In this way, the PB races in these *k* similar cases can be used as the basis for PB prediction and pacing plan recommendation. To obtain a *PB prediction* for the target runner we use the finish-times of the PB races in these *k* cases. To obtain a *pacing plan recommendation* we can use the pacing plans from the PB races. There are several ways to combine these PBs races:

1. *Best*—The simplest approach is to focus on the single *best* case (the case with the fastest finish-time) and use its PB finish-time or pacing plan directly.
2. *Mean*—Another approach is to compute the *mean* PB finish-time or the mean PB pacing plan from these *k* similar cases; we use a similarity-weighted mean for this calculation.
3. *Even*—The third option is to reuse the finish-time and pacing plan from the similar case with the most even pacing profile; this is based on the idea that even pacing is usually considered to be an optimal pacing strategy.

### Evaluation

To evaluate these ideas we use the marathon dataset from the London Marathon, using public race records from 2011–2016. Each race record includes 5km pacing and there are 5390 unique runners who have completed at least three London marathons (37% female) in this dataset—we use a minimum of three races so that we can select each runner’s PB from at least three races—leading to 12,968 unique cases; on average these runners are associated with 3.4 races.

### Methods

We implement a form of tenfold cross-validation, selecting 10% of cases to use as target/test problems to be solved and using the remaining 90% of cases as our case-base. However, since runners may be associated with multiple cases we need to ensure that the splits are constructed in such a way that runners in the 10% test sets cannot participate (via another case) in the 90% of training cases; this means that the folds are not guaranteed to be 10/90 splits, but they are close in practice.

For each target runner we use the non-PB part of their case as a query to identify the *k* most similar cases and, using the techniques described above, generate predictions and pacing plans. These are then compared to the actual PB time of the target runner and the actual pacing profile of the PB, to compute a percentage prediction error and pacing plan similarity. Lower errors mean more accurate predictions while higher pacing similarities mean closer pacing recommendations. These errors and similarities are averaged across the target runners and cross-validation folds. We compute separate averages for males and females and also an overall average for all runners.

### Results

**Fig. 9:**
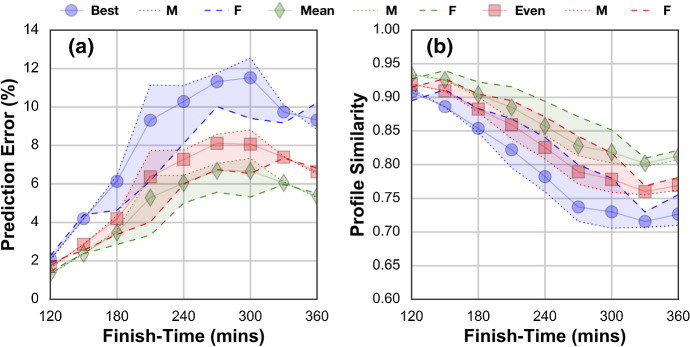
Prediction error (**a**) and pacing profile similarity (**b**) vs. *nPB* finish-time for *Best*, *Mean*, and *Even* strategies, for all runners and men and women

The results (averaged over all values of *k* between 1 and 20) are presented in Fig. 9—showing separate results for the *Best*, *Mean*, and *Even* approaches, and for males, females and all runners—and based on different PB finish-times as a useful way to see how they vary with runner ability; further results and analysis can be found in Smyth and Cunningham (2017b), Smyth and Cunningham (2018b), Smyth and Cunningham (2017a), Smyth and Cunningham (2018a).

It should be clear how, in general, prediction accuracy and pacing similarity declines for slower runners; the most accurate PB predictions and closest pacing recommendations are available to faster runners ($< 3$ hours); these more able runners are probably more experienced and run more predictable races.

It is also interesting to note how the prediction accuracy and pacing similarity are better for women than for men, regardless of *k*. This is consistent with recent research, which has shown how female runners tend to run in a more disciplined manner than their male counterparts (Trubee 2011; Deaner 2006). In short, women run more evenly paced races, they are less likely to hit the wall, and so their races unfold in a more predictable fashion. Our results suggest that this also extends to the matter of predicting a personal best time and recommending a bespoke pacing plan.

Overall the *Mean* method for combining the PBs of the *k* similar cases performs better than the *Best* and *Even* alternatives. The *Best* strategy turns out to be too optimistic, especially for higher values of *k* as it defers to the fastest PB, which is generally too fast for the target runner. Conversely, the *Mean* approach generates significantly more accurate predictions and more similar pacing recommendations for men and women, regardless of ability (PB finish-time). To place these results in context it is worth noting, as we did in Sect. 4, how they compare favourably to the current state-of-the-art in marathon time prediction, given that the mean prediction error of a variety of state-of-the-art models (many using sophisticated physiological feature) is approximately 6% while the popular Riegel prediction formula is associated with an average error of 10%; the average error for the *Mean* method is approximately 5% despite not using any training data nor having access to any physiological measures of fitness.

### Discussion and limitations

Even though this work does not have access to training or fitness data, other than past marathon race records, it has nevertheless been possible to generate reasonably accurate PB predictions and pacing plans to help runners to achieve them. In the future it will be interesting to combine this approach, using historical race times, with more recent training activities.

One immediate shortcoming of the approach discussed in this case-study is that it requires runners to have completed at least one previous marathon, which necessarily excludes first-time marathoners who are the very runners who may be most in need of pacing plans. One option is to fall-back on their experiences in shorter race distances so that, for example, cases could be constructed by mapping 10km or half-marathon races to marathon PBs, thus allowing first-timers to be matched with a similar set of cases and recommended a suitable marathon time and pacing plan. Alternatively the work of Berndsen et al. (2020b) suggests a complementary approach using ideas from collaborative filtering to suggest a suitable pacing strategy based on their current fitness level.

Another shortcoming of the approach as described is that it uses a very limited case representation, just the pacing values of a pair of non-PB and PB races, even if the runner has competed in many other marathons. Clearly there is an opportunity to include additional past races in each case if they exist. One approach to doing this was described and evaluated in Smyth and Cunningham (2018a) and led to further improvements in overall performance across all experimental conditions; similar ideas have recently been explored in sports such as speed-skating (Smyth and Willemsen 2020) and ultra-running (McConnell and Smyth 2019).

**Fig. 10:**
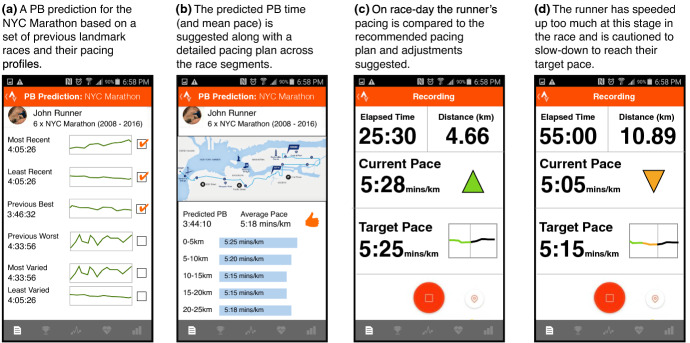
Example screens from the PB app showing the prediction/recommendation process (**a**, **b**) and the race-day feedback (**c**, **d**)

Notably, this case-study stops short of race-day. In other words, while runners may benefit from finish-time predictions and pacing plans prior to race-day, once the starter’s pistol fires they are on their own. Clearly, there is also an opportunity to support runners during their race. If things do not go to plan then inexperienced runners might be unsure about how to adjust their pacing to compensate: continuing as planned might not be wise and often the right approach is to make small but early pacing adjustments.

In Fig. 10, we present an example of a prototype app for supporting marathoners with their pre-race and in-race planning. In Fig. 10a, b the target runner selects previous races to use as the basis for PB prediction and pacing recommendation as described. Then during the race itself, progress is monitored and pacing feedback is provided in real-time. For example, in Fig. 10d we see that the runner is currently going too fast—their current pace is 5:05 mins/km but their planned pace is 5:15 mins/km—and so they are encouraged to slow-down. We have recently described and evaluated an approach for this type of in-race pacing support (Berndsen et al. 2019), by providing runners with real-time pacing suggestions at different key points in their race.

## Conclusions

For more than 20 years recommender systems have played an important and influential role in many aspects of our everyday lives, from the music and movies that entertain us to the books and news that inform us, and from the purchases we make to the vacations we take. Now, the widespread adoption of mobile computing and wearable sensors presents a new set of challenges and opportunities for recommender systems, when it comes to understanding and supporting our real-world activities and interests. With fitness, exercise, and sports applications at the leading edge of this mobile, wearable revolution, it is natural to consider such applications as novel targets for machine learning and recommender systems research.

In this work, we focus on marathon running as one such application domain. We choose it for several reasons: it is a popular and challenging sport; it is well represented by current fitness apps; it appeals to technologically savvy participants; and the sport attracts a large percentage of first-timers and novices who stand to reap significant benefits from targeted recommendations and advice. We have presented a broad vision for the different ways in which recommendation and machine learning techniques can be used to help marathon runners, particularly recreational marathon runners, who stand to benefit most from personalised recommendations and interventions to help them train more effectively and more safely. We have presented a number of concrete case studies to tackle different marathon challenges: estimating fitness levels during training; recommending tailored training sessions; predicting injury risk as training progresses; providing pacing advice to optimise finish-times. Initial evaluation results, based on large-scale, real-world datasets, have been broadly positive but now need to be tested in situ, by incorporating these ideas into systems and apps that can be used by runners as they train. This is an ongoing focus of current research.

Finally, it is worth emphasising that although the focus in this work has been on marathons and marathon runners, it should be clear that similar opportunities exist in a much wider range of sporting activities, especially other endurance sports such as cycling, triathlons, mountain biking, etc. Indeed, we have recently applied similar ideas to ultra-running (McConnell and Smyth 2019) and speed-skating (Smyth and Willemsen 2020), demonstrating similar successes to those found for marathon running, and we hope that this work will help to highlight the value that recommender systems can bring to many similar domains in the future.

## Data Availability

Some data are not available at this time as they subject to a data sharing agreement between the authors and Strava Inc. Other data, on marathon race-times, are publicly available and relevant details are provided.

## Footnotes

1 https://apps.apple.com/ie/app/couch-to-5k-runner.

2 https://www.trainingpeaks.com.

3 https://www.strava.com.

4 https://www.runkeeper.com.

5 Lactate threshold refers to the pace at which the body can no longer effectively clear lactic acid from the muscles, which is produced as a by-product of exercise; see (Billat et al. 2003; Faude et al. 2009; Poole et al. 2008). For an athlete, this means a significant increase in discomfort and it will quickly lead to the need to slow-down significantly.

6 Special thanks are due to the anonymous reviewer who suggested this particular perspective

7 https://www.virginmoneylondonmarathon.com/results/race-results

8 The pace at which a runner can no longer clear lactate is their so-called *lactate threshold*.

9 www.trainingpeaks.com

10 https://scikit-learn.org

11 Note that here $\infty$ denotes the period up until race-day.
